# Salinomycin increases chemosensitivity to the effects of doxorubicin in soft tissue sarcomas

**DOI:** 10.1186/1471-2407-13-490

**Published:** 2013-10-21

**Authors:** Sven-T Liffers, Daniel J Tilkorn, Ingo Stricker, Christoph Günter Junge, Sammy Al-Benna, Markus Vogt, Berlinda Verdoodt, Hans-U Steinau, Andrea Tannapfel, Iris Tischoff, Alireza Mirmohammadsadegh

**Affiliations:** 1 Institute of Pathology, Ruhr-University Bochum, Buerkle-de-la-Camp-Platz 1, 44789 Bochum, Germany; 2 Department of Plastic Surgery, Burn Center, Hand Surgery, Sarcoma Reference Center, BG University Hospital Bergmannsheil, Ruhr-University Bochum, 44789 Bochum, Germany

**Keywords:** Apoptosis, Salinomycin, Doxorubicin, Malignant soft tissue tumors, Chemotherapy

## Abstract

**Background:**

Chemotherapy for soft tissue sarcomas remains unsatisfactory due to their low chemosensitivity. Even the first line chemotherapeutic agent doxorubicin only yields a response rate of 18-29%. The antibiotic salinomycin, a potassium ionophore, has recently been shown to be a potent compound to deplete chemoresistant cells like cancer stem like cells (CSC) in adenocarcinomas. Here, we evaluated the effect of salinomycin on sarcoma cell lines, whereby salinomycin mono- and combination treatment with doxorubicin regimens were analyzed.

**Methods:**

To evaluate the effect of salinomycin on fibrosarcoma, rhabdomyosarcoma and liposarcoma cell lines, cells were drug exposed in single and combined treatments, respectively. The effects of the corresponding treatments were monitored by cell viability assays, cell cycle analysis, caspase 3/7 and 9 activity assays. Further we analyzed NF-κB activity; p53, p21 and PUMA transcription levels, together with p53 expression and serine 15 phosphorylation.

**Results:**

The combination of salinomycin with doxorubicin enhanced caspase activation and increased the sub-G1 fraction. The combined treatment yielded higher NF-κB activity, and *p53, p21* and *PUMA* transcription, whereas the salinomycin monotreatment did not cause any significant changes.

**Conclusions:**

Salinomycin increases the chemosensitivity of sarcoma cell lines - even at sub-lethal concentrations - to the cytostatic drug doxorubicin. These findings support a strategy to decrease the doxorubicin concentration in combination with salinomycin in order to reduce toxic side effects.

## Background

Soft tissue sarcomas are a rare and heterogeneous entity of tumors with an annual incidence of 2-4/100,000 [1]. In contrast to carcinomas, soft tissue tumors are of mesenchymal origin. According to the tumor grading the 5-year survival ranges from 72-83% in well differentiated G1 sarcomas, 53-59% in G2 sarcomas to 26-42% in G3 sarcomas [2]. Metastatic disease becomes evident within the first 2–3 years after initial diagnosis and is the main cause of mortality in these patients [3]. Despite multi-disciplinary treatment (i.e. surgery, chemotherapy and radiation therapy), the rate of recurrence remains higher than 50%, and results in diffuse metastatic disease and the death of the patients [4]. Chemotherapy for advanced tumors remains unsatisfactory due to low chemosensitivity despite combination chemotherapeutics. The most effective chemotherapeutic agents are the anthracyclines doxorubicin and epirubicin [5-7]. Doxorubicin still remains the first line chemotherapeutic for soft tissue sarcomas. Unfortunately, its cytostatic effect in therapeutic doses is frequently insufficient (monotherapy response rate of only 18-29%); but the use of higher doxorubicin doses is limited by the development of systemic toxicity, especially cardiotoxicity. In addition to the poor response rate to doxorubicin, the development of drug resistance remain an unresolved problem [8-12].

The anionic and weakly acidic antibiotic salinomycin acts in different biological membranes including cytoplasmic and mitochondrial membranes. In addition to its antimicrobial properties, salinomycin selectively depletes breast cancer stem cells from tumorspheres and impedes breast tumor growth in mice xenograft experiments [13]. The activation of apoptotic pathways by salinomycin is independent of p53 and caspase activation [14]. Further, it has been reported that salinomycin sensitized cancer cells by reducing p21 levels [15,16]. However, little is known about its impact on sarcomas.

In order to improve the oncological treatment of patients with soft tissue sarcoma mechanisms to overcome drug resistance and reduce drug toxicity must be identified. Therefore, the aim of this study was to evaluate the effect of salinomycin on the chemosensitivity to doxorubicin in three different soft tissue sarcoma cell lines.

## Methods

### Cell culture

The human soft tissue sarcoma cell lines SW872 (liposarcoma cell line), A204 (rhabdomyosarcoma cell line) and HT-1080 (fibrosarcoma cell linie) (ATCC-LGC Standards, Wesel, Germany) were cultured in DMEM (PAN-Biotech, Aidenbach, Germany) media containing 10% fetal bovine serum (Hyclone-Thermo Scientific, Bonn, Germany) and 1% penicillin/streptomycin (PAN-Biotech, Aidenbach, Germany) and were incubated at 37°C and 5% CO_2_.

### Cell viability assay

Cells were seeded at 2×10^4^ per well in 24 well plates and treated 16 h later at the indicated drug concentrations. Forty-eight hours after the application of salinomycin (Sigma, Taufkirchen, Germany), doxorubicin (Sigma, Taufkirchen, Germany) or both compounds together, respectively, 100 μl of MTT solution (5 mg/mL) was added per well. Cells were lysed with 250 μl triplex solution (1 mM HCl; 5% iso-butanol; 1% SDS), after 3 h of incubation. Optical density was measured at 562 nm with a background correction at 630 nm. All data points were normalized with respect to the DMSO control.

### Cytotoxicity assay

Alternatively, determination of cytotoxicity was carried out using the MultiTox-Glo assay (Promega, Mannheim, Germany). For this, 3000 cells per well were seeded in 96 well plates (Corning, Amsterdam, Netherlands), and treated 16 h after cell seeding in the presence of a dose–response of doxorubicin in the presence and absence of 1 μM Salinomycin (i.e. 751 ng/mL). Toxicity was measured after 24 h according to the manufacturer’s instructions in four independent measurements. The cytotoxicity is expressed as the ratio of live cell fluorescence to dead cell luminescence, relative to the vehicle control.

### Caspase assay

Caspase activities were measured using the Caspase Glo 3/7 and 9 assays from Promega (Mannheim, Germany), according to the manufacturer’s instructions. The resulting luminescence was measured with a Tecan M200 microplate reader (Tecan, Crailsheim, Germany) for 10 s measurement period. Values were corrected for differences in cell numbers by simultaneously conducting a MTT assay (Carl Roth, Karlsruhe, Germany).

### Annexin V analysis

Cells were plated at 1.5×10^5^ cells per well in a 6 well plate 16 h before treatment and treated as indicated. DMSO served as a vehicle control. Twenty-four hours after treatment, cells were harvested and apoptosis was determined by Annexin V-Alexa 488 (Life Technologies, Darmstadt, Germany) staining. Cells were counterstained with 0.2 μM TO-PRO-3 iodide (Life Technologies, Darmstadt, Germany) to discriminate between vital and dead cells. After staining the cells were measured on a Guava HT flow cytometer (Millipore, Schwalbach am Taunus, Germany) in triplicates. Data analysis was carried out using the Express Pro 2.0 software (Millipore, Schwalbach am Taunus, Germany).

### Cell cycle analysis

Cells were plated at 1.5×10^5^ cells per well in a 6 well plate 16 h before treatment. Cells were trypsinized, collected by centrifugation, and fixed in ice-cold 70% ethanol, 48 h post treatment. Fixed cells were collected by centrifugation and washed once with PBS. Subsequently, the cells were stained in PBS buffer containing 50 μg/mL propidium iodide (MP Biochemicals, Illkirch, France), 0.1% Triton X-100 (Sigma, Taufkirchen, Germany) and 10 U/mL RNase A (Sigma, Taufkirchen, Germany). After incubation for 30 min at 37°C, cell cycle profiles were measured on a Guava HT flow cytometer following data analysis using Express Pro 2.0.

### Reporter NF-κB analysis

NF-κB activity was analyzed in triplicates by transfecting 5×10^4^ HT-1080 cells with 300 ng pGL4.32[luc2P/NF-kB-RE/Hygro] vector (Promega, Mannheim, Germany), using X-tremeGENE (Roche, Mannheim, Germany); 10 ng pRL-TK (Promega, Mannheim, Germany) served as transfection control. Firefly and *Renilla* luciferase activities were measured 6 h and 10 h post treatment. The luciferase-signals were measured for 10s (Tecan M2000, Crailsheim, Germany). The *Renilla* signal was used for normalization. Mean values and SEM were calculated from triplicates.

### Western blot analysis

HT-1080 cells were seeded with 1×10^6^ cells per 10 cm dish. Sixteen hours post seeding, the cells were subjected for 6 h to the different treatments. The isolation of nuclear and cytoplasmic fractions were carried out after cells were allowed to swell on ice for 10 min in 500 μl of hypotonic buffer (20 mM Tris–HCl, pH 7.4, 5 mM MgCl_2_, 1.5 mM KCl, 0.1% NP-40, 50 mM NaF, 2 mM sodium orthovanadate, and protease inhibitors (Complete, Roche)). Cells were subsequently disrupted by passing them several times through a 26 ½ gauge syringe needle, followed by a centrifugation at 800×g (5 min; 4°C). The supernatants were further centrifuged at 10,000×g (15 min; 4°C) to remove insoluble pellets, and the resulting supernatants were collected as the cytoplasmic fractions. The pellets were resuspended in 100 μl of TKM buffer (20 mM Tris-acetate; pH 7.4, 50 mM KCl, 5 mM MgCl_2_, containing protease and phosphatase inhibitors). After centrifugation (800×g; 10 min; 4°C), the supernatants were collected like the cytoplasmic fractions. From each fraction, 30 μg total protein were subjected to 4-12% BisTris-PAGE and transferred onto PVDF membranes (Millipore, Schwalbach, Germany) with 2 mA/cm^2^ for 1 h. After protein transfer membranes were blocked in PBS-T containing 5% (w/v) skimmed milk, for 1 h and incubated with anti-pS15 p53 antibody (Cell Signaling, Frankfurt am Main, Germany) and anti-p53 (Clone DO-1, Sigma-Aldrich, Taufkirchen, Germany) overnight (1:1000 in PBS-T). As loading control for the cytoplasmic fraction, anti-α-tubulin antibody (Sigma-Aldrich, Taufkirchen, Germany) was used at 1:2500 dilution in PBS-T for 1 h at room temperature whereas anti-lamin (Cell Signaling, Frankfurt am Main, Germany) at 1:1000 served as loading control for the nuclear fraction. Membranes were incubated for detection with secondary antibodies raised against rabbit labeled with CyDye800 (Licor, Bad Homburg, Germany) and mouse labeled with CyDye700 (Licor, Bad Homburg, Germany) for 1 h at room temperature. Signals were detected by Odyssee Scanner (Licor, Bad Homburg, Germany).

### RNA isolation and RT-PCR

RNA was isolated using the RNeasy mini kit (Qiagen, Hilden, Germany), according to the manufacturer’s instructions. To remove possible genomic contamination, DNA digestion was performed by using the Ambion TurboDNAse purification kit (Life Technologies, Darmstadt, Germany) as described in the kit’s manual. The RNA concentration was measured with a Tecan M200 (Tecan, Crailsheim, Germany). For quantitative reverse transcriptase-polymerase chain reaction (qRT-PCR), first-strand cDNA was synthesized from 1 μg of total RNA using the Applied Biosystems High Capacity cDNA reverse transcription kit (Life Technologies, Darmstadt, Germany). cDNA was amplified on an Eppendorf Realplex4 thermal cycler (Hamburg, Germany) using Promega GoTaq qPCR Master Mix. The sequence for the PCR primers are: *p21*: 5′-GGCGGCAGACCAGCATGACAGATT-3′ and 5′-GCAGGGGGCGGCCAGGGTAT-3′; p53: 5′-CTAAGCGAGCACTGCCCAAC-3′ and 5′-GGCCTCATTCAGCTCTCGGA-3′; actin: 5′-CATGCCATCCTGCGTCTGGACC-3′ and 5′-ACATGGTGGTGCCGCCAGACAG-3′, whereas PUMA expression was analyzed using the QuantiTect *Primer* Assay (Qiagen, Hilden, Germany). After an initial activation at 94°C for 3 min, 40 cycles of 94°C for 15 s, 55°C for 30 seconds, and 72°C for 45 s. Experiments were done in triplicates and fold changes calculated based on the ∆∆Ct method.

### Statistics

Significance testing between pairs of treatments was done by unpaired two tailed Student’s *t*-test using Welch’s correction if the F-test indicated a significant difference between variances. Differences were considered significant if *p* < 0.05. The IC_50_ was estimated from the MTT absorbance data, using the four parameter logistic model in R 2.15.1. The synergy between the effects of salinomycin and Doxorubicin was determined by the combination index (CI) based on IC_50_ isobologram [17]. A CI < 0.9 was considered as synergism between the two compounds whereas 0.9 – 1.1 indicates an additive effect.

## Results

### Impact of salinomycin on different soft tissue sarcoma cell lines

The heterogeneity of soft tissue sarcomas led us to investigate the impact of salinomycin on soft tissue sarcoma cell lines of different origin. The assessed cell line panel consists of the rhabdomyosarcoma cell line A204, the fibrosarcoma cell line HT-1080, and the liposarcoma cell line SW872.

**Figure 1:**
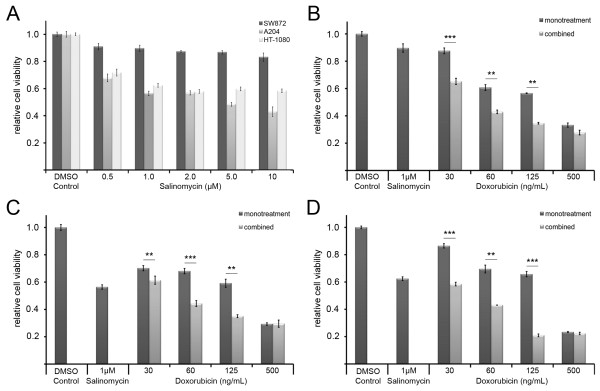
**Dose dependent changes in the viability of soft tissue sarcoma cells in the presence of salinomycin, doxorubicin, and the combined treatment.** The impact of salinomycin was analyzed at different concentrations as indicated **(A)**. Cell viability of adherent growing SW872 **(B)**, A204 **(C)** and HT-1080 **(D)** cells was measured by MTT assay 48 h post doxorubicin treatment. Error bars denote the standard error (n = 3). Student’s *t-test* was conducted to determine the significance (* *p* < 0.05, ** *p* < 0.01, *** *p* < 0.001).

The dose response experiment with salinomycin revealed different reductions in the growth activity of the analyzed soft tissue sarcoma cell lines A204, HT-1080 and SW872. The strongest decrease in cell viability was with the A204 cells which decreased from 67.8% to 43.0%. The HT-1080 displayed a reduction to 60% for all concentrations, except 0.5 μM. The SW872 cells showed less sensitivity to salinomycin treatment, with a mild reduction in cell viability ranging from 9.1% in the presence of 0.5 μM salinomycin to 16.9% for 10 μM salinomycin (Figure 1A).

### Impact of doxorubicin treatment on different soft tissue sarcoma cell lines

The dose response experiment for the doxorubicin monotherapy comprised of treatments with 30 ng/mL, 60 ng/mL, 125 ng/mL or 500 ng/mL doxorubicin. In contrast to the salinomycin treatment alone, a dose response was observed for all cell lines in the presence of doxorubicin (Figure 1B-D).

### Impact of the combined salinomycin and doxorubicin treatment on different soft tissue sarcoma cell lines

**Figure 2:**
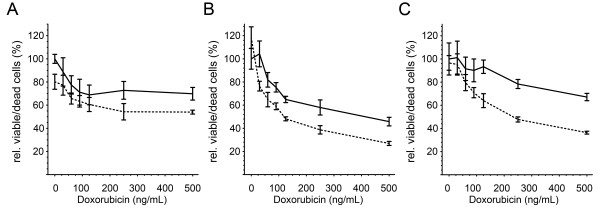
**Salinomycin affects the cytotoxicity of doxorubicin.** Changes on cell viability were analyzed by the cytotoxicity ratio (viable/dead cells) for SW872 **(A)**, A204 **(B)** and HT-1080 **(C)** cells 24 h post treatment. The solid line indicates the doxorubicin monotreatment and the dashed the combined treatment of doxorubicin with 1 μM salinomycin. Error bars denote the standard error (n = 4). Student’s *t-test* was conducted to determine the significance (* *p* < 0.05, ** *p* < 0.01, *** *p* < 0.001).

**Figure 3:**
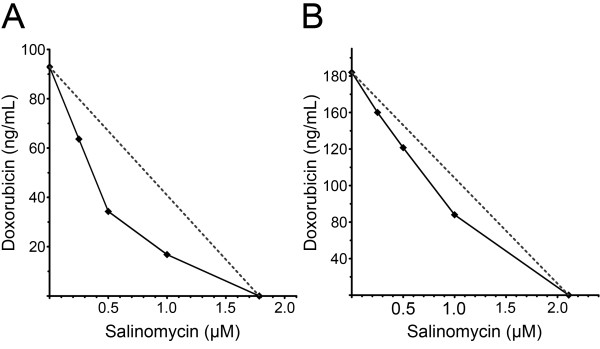
**Salinomycin and doxorubicin synergistically effects the treatment of soft tissue sarcoma cells.** Isobologram analysis of A204 **(A)** and HT-1080 **(B)** cells for doxorubicin in combination with salinomycin based on the IC_50_.

The combined treatment with 1 μM salinomycin and different doxorubicin concentrations (30 ng/mL, 60 ng/mL, 125 ng/mL and 500 ng/mL, respectively) revealed on average a 1.5 fold decrease of the relative cell growth activity in comparison to doxorubicin alone. The most significant change was detected for HT-1080 cells at a concentration of 125 ng/mL doxorubicin in combination with 1 μM salinomycin, which revealed a 3-fold decrease in cell viability compared to the corresponding doxorubicin monotherapy (Figure 1B-D). In summary, the combined treatment of 125 ng/mL doxorubicin with a sub-lethal concentration of 1 μM salinomycin led to a reduction of cell viability equal to the 4 times higher doxorubicin monotherapy, whereas the increase of the salinomycin concentration to 2 μM did not lead to a further decrease in cell viability (data not shown). To further support the observed synergistic effect between doxorubicin and salinomycin we analyzed the ratio between viable and dead cells by cytotoxicity assay (Figure 2). The ratio of viable and dead cells in the presence of a doxorubicin dose response in the presence and absence of salinomycin revealed for A204 and HT-1080 cells a significant increase at a doxorubicin concentration of 125 ng/mL and higher for the combined treatment. Having shown that the concomitant administration of doxorubicin and salinomycin leads to a better response of the analyzed cell lines in two independent assays, we analyzed the potential synergistic effects by isobologram analysis based on the IC_50_. The weak response of SW872 cells to the salinomycin monotreatment does not allow for the estimation of an IC_50_ for this cell line. Therefore, we excluded the SW872 cells from the isobologram analysis. For A204 and HT-1080 cells a synergistic effect between doxorubicin and salinomycin (Figure 3) was observed. The calculation of the combination index (CI) revealed that 1 μM salinomycin synergistically cooperates with doxorubicin to decrease cell viability in both cell lines HT-1080 (CI = 0.84) and A204 cells (CI = 0.74). A salinomycin dose below 1 μM leads only in A204 cells to a synergistic effect with doxorubicin.

### Effects of salinomycin and doxorubicin on apoptosis

**Figure 4:**
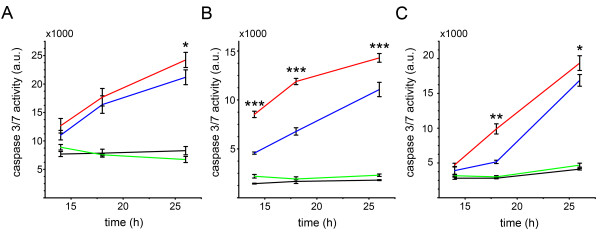
**The combined treatment of doxorubicin and salinomycin activates caspase dependent apoptosis. ** Caspase assays were conducted 14 h, 18 h and 26 h after drug administration for SW872 **(A)**, A204 **(B)** and HT-1080 **(C)** cells. The soft tissue sarcoma cells were cultivated with DMSO (black line), 1 μM salinomycin (green line), 125 ng/mL doxorubicin (blue line) and the combination of 1 μM salinomycin with 125 ng/mL doxorubicin (red line). Error bars denote the standard error (n = 3). (* *p* < 0.05, ** *p* < 0.01, *** *p* < 0.001).

Given the synergistic effect of salinomycin and doxorubicin on the investigated cell lines, we further elucidated if the observed cell death was apoptosis related. Therefore, we analyzed the activity of the caspases 3/7 as an indicator for the activation of caspase dependent apoptosis. Additionally, we analyzed the early apoptotic response of soft tissue sarcoma cells by Annexin V staining, and the sub-G1-fraction as a late event in apoptosis. The caspase 3/7 induction was analyzed 14 h, 18 h, and 26 h post treatment by luminometric measurements (Figure 4A-C). During the whole time course, no induction of caspase 3/7 activity was detectable in any of the analyzed cell lines for salinomycin monotherapy, whereas doxorubicin induced caspase 3/7 in a cell line dependent manner. The combined treatment (i.e. 1 μM salinomycin and 125 ng/mL doxorubicin) resulted in a significant increase in the caspase 3/7 activity.

**Figure 5:**
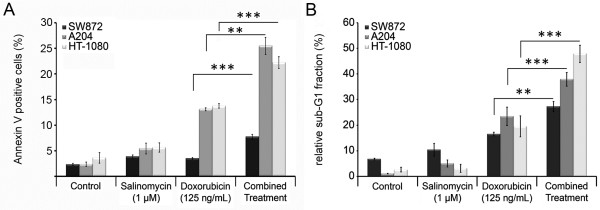
**Salinomycin sensitizes soft tissue sarcoma cells to doxorubicin induced apoptosis.** Each cell line was cultivated in the presence of salinomycin (1 μM); doxorubicin (125 ng/mL), or with a combination of both compounds before cells were subjected to analysis. Early apoptosis events were analyzed by Annexin V^+^ TO-PRO-3^-^ cell, 24 h post treatment **(A)**. DNA fragmentation was measured by flow cytometry as a sub-G1 cell fraction after propidium iodide staining, 48 h post treatment **(B)**. Error bars denote the standard error (n = 3). (* *p* < 0.05, ** *p* < 0.01, *** *p* < 0.001).

Next we analyzed the Annexin V^+^ TO-PRO-3^-^ cell fraction as an indicator of early apoptotic cells 24 h post treatment (Figure 5A). The assessed cell lines showed the highest amount of Annexin V^+^ TO-PRO-3^-^ cells after the combined treatment. The observed increase of this cell fraction was significantly higher compared to the corresponding single treatments. In concordance with the cell viability data (Figures 1 and 2) and the annexin V staining HT-1080 and A204 cells are the most sensitive cell lines in the flow cytometric analysis. The smallest change in the sub-G1 fraction was observed for SW872 cells, which showed an increase in the sub-G1 fraction of 10.7% (doxorubicin monotherapy vs. combined treatment; p = 0.0052), followed by A204 cells which showed an increase of 14.4% (p = 0.00072). The highest increase in the sub-G1 fraction with 28.3% was noted for the HT-1080 cells (p = 0.00088) (Figure 5B). Our flow cytometric analysis confirmed that the reduction noted in the cell viability data for the doxorubicin monotherapy and the combination therapy related to an increased apoptosis rate. On the other hand, no significant increase in the sub-G1 fraction was detected after 48 h of salinomycin treatment in two of the three analyzed cell lines. This indicates that in contrast to the treatment including doxorubicin, the reduction in the cell viability assay was not associated with apoptosis of the sarcoma cells.

### Combined treatment enhances p53 signaling in HT-1080 cells

**Figure 6:**
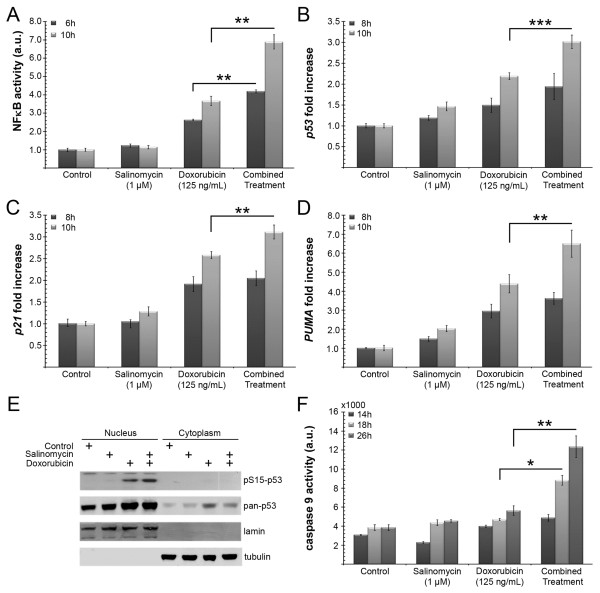
**Impact of the combined treatment on NF-κB activity and p53 mediated apoptosis.** NF-κB reporter activities in HT-1080 cells, 6 h and 10 h post-treatment as indicated. The firefly reporter activities were normalized to Renilla luciferase **(A)**. Real-time RT-PCR analysis of p53 **(B)**, p21 **(C)** and PUMA **(D)** expression in HT-1080 cells following treatment for 8 h and 10 h. Actin was used for normalization. Western blot analysis of p53 serine 15 phosphorylation 6 h after treatment **(E)**. Lamin served as loading control for the nuclear, and tubulin for the cytoplasmic fraction. Time course of caspase 9 activity 14 h, 18 h, 26 h after treatment as indicated **(F)**. (* *p* < 0.05, ** *p* < 0.01, *** *p* < 0.001).

HT-1080 cells are known for their NF-κB-mediated chemoresistance in response to doxorubicin [18]. Hence we focused on the regulation of NF-κB activity according to the analyzed treatment regimens. In accordance with the caspase assay and the sub-G1 analysis, no significant changes in NF-κB activity were observed in the presence of salinomycin alone (Figure 6A). The presence of doxorubicin resulted in an increase of NF-κB activity (2.6 fold increase after 6 h and 3.7 fold increase after 10 h). Furthermore, the combined treatment resulted in a significantly higher NF-κB activation (i.e. 4.2 fold increase after 6 h and 6.9 fold increase after 10 h), compared to doxorubicin alone.

NF-κB has been shown to be important for the activation of p53 transcription by several agents [19,20]. These reports in combination with the observed increased caspase 3/7 activities in co-treated cells (Figure 4), opted us to investigate the p53 mediated pro-apoptotic effect of NF-κB. Therefore *p53* mRNA levels were analyzed at 8 and 10 h post treatment by qRT-PCR (Figure 6B). A three times higher increase of the *p53* level was detected compared to the control group, whereas the doxorubicin monotreatment led to a 2.2 fold-increase. To test if the elevated *p53* expression correlates with the transcription of p53 target genes, the expression of *p21*, a canonical p53 target, and *PUMA,* a pro-apoptotic gene of which the transcription depends on p53 and NF-κB, were analyzed. A 2.6 fold increase of *p21* expression for the doxorubicin arm was detected versus a 3.1 fold increase for the combined treatment option (Figure 6C). After 10 h *PUMA* transcription was upregulated 6.5 fold in the combined treatment group, whereas doxorubicin alone led only to a 4.4 fold-increase (Figure 6D). Therefore, each of the p53 target genes showed a time dependent increase at the transcription level. The salinomycin monotreatment did not reveal any fold changes exceeding a factor of 2 (Figure 6B-D). In addition to the changes of the transcription level, we investigated the serine 15 phosphorylation of p53 as an indicator of genotoxic stress. The phosphorylation state of serine 15 was analyzed 6 h post treatment. The Western blot analysis clearly demonstrated the accumulation of p53 phospho-serine 15 in the nuclear extracts of HT-1080 cells which were subjected to either doxorubicin or combined treatment. In addition the extracts of the combined treatment displayed a stronger serine 15 phosphorylation (Figure 6E). In the presence of salinomycin, doxorubicin further enhanced the phosphorylation of p53 at serine 15. This supports the hypothesis that salinomycin sensitizes soft tissue sarcoma cells to the effects of doxorubicin, rather than inducing apoptosis on its own because salinomycin was not able to induce serine 15 phosphorylation in the absence of the genotoxic agent doxorubicin.

Finally, we analyzed the caspase 9 activation of the different treatment regimes. Caspase 9 activation is initiated by PUMA which inhibits Bcl-2. The caspase 9 activity was significantly upregulated after 18 h, and further increased after 26 h for the combined versus the doxorubicin single treatment (Figure 6F) which correlates well with the Caspase 3/7 activities (Figure 4C).

## Discussion

Malignant soft tissue tumors are composed of a heterogeneous cell population which exhibits varying degrees of chemosensitivity. A high rate of recurrence would be expected even if only a minor percentage of the cancer cells with a high resistance to systemic therapy persist in the patient [21]. This hypothesis is also reflected by the clinical characteristics of these tumors with a marked chemoresistance, high rate of relapse and metastasis. The antibiotic salinomycin has been demonstrated to overcome drug resistance in various apoptosis-resistant human cancer cells including CSCs [13,22,23]. Furthermore, it has been shown that the cell death induced by salinomycin occurs independent of p53 and caspase activation [14,24], pathways that are frequently disturbed in tumors. Therefore, we studied whether salinomycin could have a therapeutic use in sarcomas.

The sarcoma cell lines HT-1080, A204, and SW872 displayed a slight reduction of the cell growth in MTT assays between 0.5 μM to 10 μM, without any significant changes at concentrations higher than 1 μM, but further analysis revealed no increases in caspase 3/7 activity or the sub-G1 fraction and only a minor increase in Annexin V staining. These results indicated that at the tested concentration of 1 μM salinomycin no apoptotic cellular response occurred. In contrast, we were able to show that even at low salinomycin doses, which did not directly provoke cell death, salinomycin was able to enhance the cellular response to doxorubicin. The concurrent administration of 1 μM salinomycin in combination with doxorubicin doses ranging from 30 ng/mL to 500 ng/mL showed a synergistic effect on apoptosis. This was supported by the lowered doxorubicin IC_50_ in all cell lines in the presence of salinomycin. The salinomycin concentration in the present study was 10–20 fold below the concentrations used in a previously published study [15], and below the direct toxic dose of salinomycin for the analyzed sarcoma cell lines. These findings further suggest that salinomycin is acting synergistically to doxorubicin therapy even if used at a sublethal concentration. This proved that the combination is more effective in the treatment of sarcoma cells than the doxorubicin monotherapy on its own.

The development of multiple mechanisms of drug and apoptosis resistance is a hallmark of soft tissue sarcomas. For sarcoma cell lines the abrogation of p53-induced apoptosis by blocking NF-κB is described as a mechanism of drug resistance [25,26], whereas HT-1080 cells acquire chemoresistance through the activation of NF-κB to mediate cell survival [18]. This demonstrates the dual function of NF-κB in the regulation of pro- and anti-apoptotic cellular responses. In this study we observed that the activity of NF-κB is higher in cells which were simultaneously treated with doxorubicin and salinomycin than in the doxorubicin monotherapy. Therefore we propose that in the presence of 1 μM salinomycin, the NF-κB signaling induced by doxorubicin is shifted towards a pro-apoptotic response rather than cell survival in HT-1080 cells. NF-κB has been proposed to be a transcription factor of p53 [27,28]. This led us to analyze the canonical p53 targets PUMA and p21. PUMA plays a pivotal role in p53 dependent and independent apoptosis. Normally expressed at low concentrations, PUMA is markedly induced following DNA damage. This is further supported by the strong increase of PUMA at the transcription level 10 h after drug administration, whereas the p21 induction is only slightly different from doxorubicin monotreatment. In addition to the p53 dependent induction, Wang *et al*. reported that PUMA expression can also be induced directly by NF-κB independent of p53 signaling [29]. The significant increase of PUMA expression by the combined treatment is further supported by the stronger activation of caspase 9, a canonical downstream effector of PUMA. Therefore our data support a model in which a sub-lethal dose of salinomycin in combination with doxorubicin enables the pro-apoptotic function of NF-κB and enhances the activation of PUMA-mediated apoptosis. Furthermore, Liu *et al.* reported that PUMA is also important in p53-dependent apoptosis for the depletion of adult stem cells [30].

## Conclusion

Now that salinomycin has been shown to be efficient in soft tissue sarcomas *in vitro*, the study of its safety, toxicity, pharmacology and anticancer activity *in vivo* will be the next step. In conclusion, this study demonstrated that salinomycin increases the potency of doxorubicin therapy on sarcoma cell lines and permits lower-dose doxorubicin therapy potentially reducing the development of its systemic toxicity. Salinomycin may be a valuable chemotherapeutic adjuvant in the treatment of soft tissue sarcoma patients.

## Competing interests

All authors declare that they have no competing interest in regard to this manuscript.

## Authors’ contributions

STL, DJT, IS, IT and AM conceived and designed the experiments. STL, DJT, CGJ, BV and MV performed the experimental work. STL, DJT, IS, BV, IT and AM participate in data analysis and interpretation. SAB, HUS and AT revised the manuscript critically for important intellectual content. All authors read and approved the final manuscript.

## Pre-publication history

The pre-publication history for this paper can be accessed here:

http://www.biomedcentral.com/1471-2407/13/490/prepub
